# Development of a Multifunctional Benzophenone Linker for Peptide Stapling and Photoaffinity Labelling

**DOI:** 10.1002/cbic.201500648

**Published:** 2016-03-16

**Authors:** Yuteng Wu, Lasse B. Olsen, Yu Heng Lau, Claus Hatt Jensen, Maxim Rossmann, Ysobel R. Baker, Hannah F. Sore, Súil Collins, David R. Spring

**Affiliations:** ^1^ University Chemical LaboratoryUniversity of CambridgeLensfield RoadCambridgeCB2 1EWUK; ^2^ Department of BiochemistryUniversity of Cambridge80 Tennis Court RoadCambridgeCB2 1GAUK

**Keywords:** click chemistry, linker, MDM2, photoaffinity labeling, stapled peptide

## Abstract

Photoaffinity labelling is a useful method for studying how proteins interact with ligands and biomolecules, and can help identify and characterise new targets for the development of new therapeutics. We present the design and synthesis of a novel multifunctional benzophenone linker that serves as both a photo‐crosslinking motif and a peptide stapling reagent. Using double‐click stapling, we attached the benzophenone to the peptide via the staple linker, rather than by modifying the peptide sequence with a photo‐crosslinking amino acid. When applied to a p53‐derived peptide, the resulting photoreactive stapled peptide was able to preferentially crosslink with MDM2 in the presence of competing protein. This multifunctional linker also features an extra alkyne handle for downstream applications such as pull‐down assays, and can be used to investigate the target selectivity of stapled peptides.

Protein–protein interactions (PPIs) are inextricably involved in a host of cellular functions, with aberrant activity linked to a variety of human diseases.^1^ Tool compounds that can probe specific PPIs are vital for unravelling the functions of individual proteins within a complex protein network, and can potentially identify and characterise new targets for drug development.^2^ In particular, compounds that can be used for photoaffinity labelling are powerful tools for studying the interactions of proteins with ligands or other biomolecules.^3^ Photoaffinity labelling involves the use of a photoactivatable functionality that, on exposure to UV light, can form a covalent linkage to biomolecules within close proximity.^4^ This irreversible process enables subsequent analysis of the interaction.^5^

As part of our work on stapled peptides to inhibit PPIs, we were interested in developing photoaffinity tools to characterise our peptides and their target PPIs. In peptide stapling, a promising strategy for designing α‐helix mimetic inhibitors, two amino acid side‐chains are joined to form a macrocycle.^6^ Non‐proteogenic amino acids are commonly used for macrocylisation; however, techniques involving all native residues are also available.^7^ The resulting peptide is stabilised in a helical conformation, which can lead to improved binding affinity and pharmacokinetic properties, relative to those of the linear analogues.^8^ Walensky and co‐workers used photoaffinity probes to covalently trap proteins in the BH3/BCL2 complex by incorporating unnatural benzophenone‐bearing amino acids into hydrocarbon‐stapled BH3 peptides.^9^

We have previously reported a double‐click stapling technique where the peptide and linker are two separate components. This allowed facile modification of the linker to tailor the reactivity of the overall stapled peptide.8b, 10

In this study, we sought to incorporate a photoactive benzophenone moiety into the stapling linker itself. By installing the photoactive group in the linker the peptide sequence is not modified, thus possibly minimising disruption of the overall binding affinity. We reasoned that combining double‐click stapling groups with photoaffinity labelling within one linker could provide a rapid means of assessing the target selectivity of stapled peptides as well as the identification of potential off‐target interactions. In addition to the double‐click stapling motif and photoactivatable group, we also designed an extra protected alkyne handle for downstream applications such as pull‐down assays (Scheme 1). As a proof of concept, we applied our photolabelling linker to a p53 peptide for binding MDM2, a PPI that has received significant attention in anti‐cancer therapeutics. Overexpression of the E3 ubiquitin ligase MDM2 in some cancer cell lines leads to loss of function of p53, a crucial tumour suppressor protein.^11^ This in turn can result in uncontrolled abnormal cell growth and subsequent cancer progression.

**Scheme 1:**
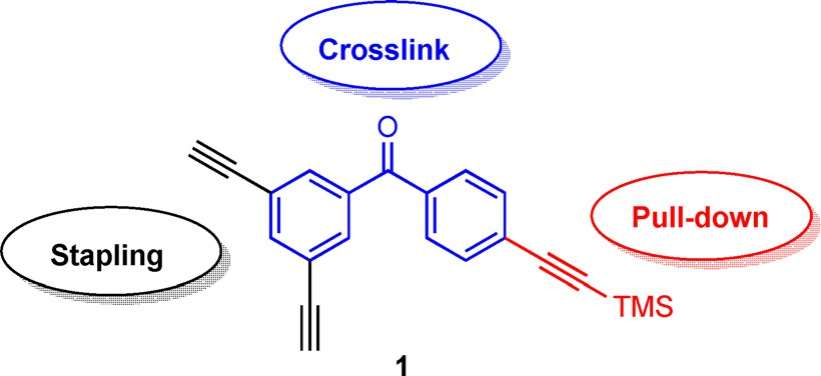
Multifunctional linker with two alkynes for double‐click stapling (black), a benzophenone group for photo‐crosslinking (blue), and an additional protected alkyne handle for pull‐down assays (red).

The novel benzophenone linker **1** was synthesised in four steps (Scheme 2), commencing with a Sonogashira coupling of (commercially available) 3,5‐dibromobenzaldehyde (**2**) with trimethylsilylacetylene to give the bis‐TMS‐protected intermediate **3** in good yield. Deprotection of the acetylene groups under basic conditions resulted in the dialkyne intermediate **4**. Nucleophilic addition with pre‐prepared (4‐((trimethylsilyl)ethylnyl)phenyl)lithium reagent afforded the secondary alcohol **6**. Oxidation of the alcohol with Dess–Martin periodinane (DMP) gave the final dialkynyl benzophenone linker **1** in an overall yield of 12 % for the four‐step synthesis.

**Scheme 2:**
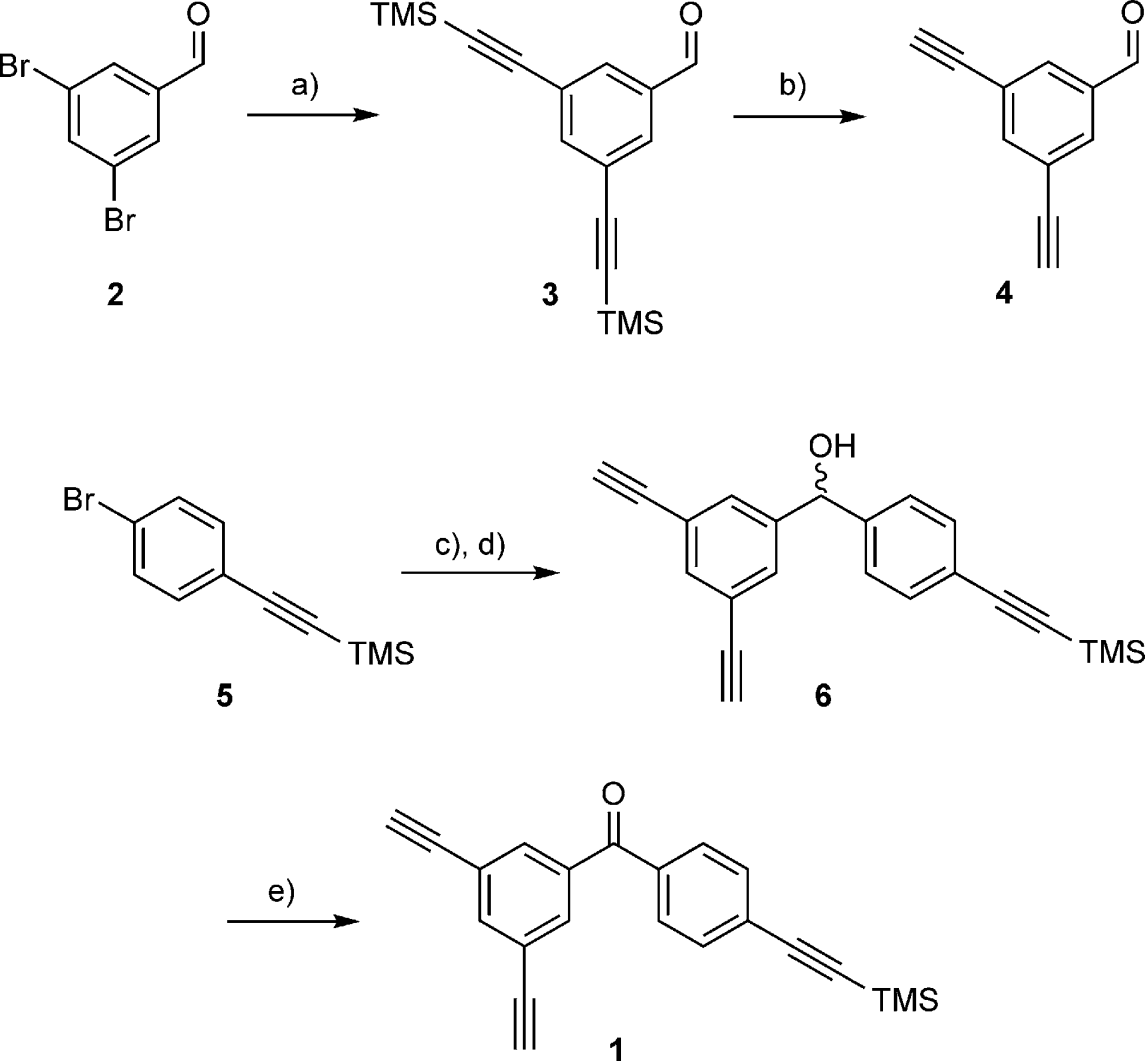
Four‐step synthesis of benzophenone stapling linker **1**. DMP: Dess–Martin periodinane. a) TMS‐C≡CH, Pd(PPh_3_)_4_, CuI, Et_3_N, THF, RT, 24 h (76 %); b) NaOH, THF, 0 °C, 3 h (81 %); c) *n*BuLi; d) **4**, THF, −78 °C, 16 h (31 % over two steps); e) DMP, CH_2_Cl_2_, RT, 2 h (64 %).

In order to synthesise the photoactive stapled peptide, we followed our previously reported, optimised copper‐catalysed double‐click method with a peptide sequence (**A0**) derived from an alpha‐helix in the N‐terminal transactivation domain of p53.8b, 10, 12 This approach involved the installation of azide‐containing non‐natural amino acids at defined positions (*i*, *i*+7) within the peptide sequence. These azide functionalities were then reacted with the bis‐alkyne‐benzophenone linking unit **1** by copper catalysis. Formation of the TMS‐protected stapled peptide product was seen initially (Figure S2 in the Supporting Information). Subsequently, full TMS deprotection was observed after six hours under the click‐reaction conditions to yield the desired photoaffinity probe **A1** in one step (Scheme 3, Table 1).

**Scheme 3:**
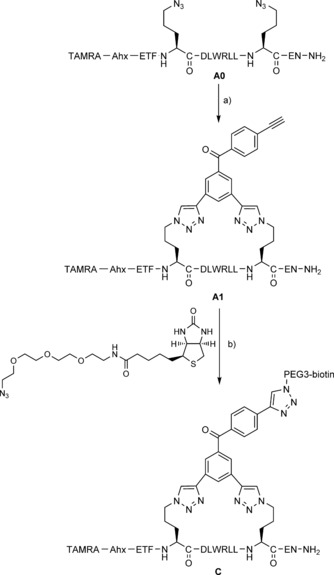
Copper‐catalysed double‐click stapling and attachment of a biotin‐PEG3 pull‐down handle onto the resulting stapled peptide. a) **1**, CuSO_4_ **⋅**5 H_2_O, THPTA, sodium l‐ascorbate, *t*BuOH/H_2_O (1:1), RT, 6 h (46 %); b) CuSO_4_ **⋅**5 H_2_O, THPTA, sodium l‐ascorbate, *t*BuOH/H_2_O (1:1), RT, 6 h (full conversion by HPLC).

**Table 1:** In vitro binding affinity of photoactive stapled peptides for MDM2 by isothermal calorimetry.

Peptide	Sequence	*K*_d_ [nm]
**A1**	TAMRA‐Ahx‐ETF‐Orn(N_3_)‐DLWRLL‐Orn(N_3_)‐EN‐NH_2_	18±6
**B1(F3A)**	TAMRA‐Ahx‐ETA‐Orn(N_3_)‐DLWRLL‐Orn(N_3_)‐EN‐NH_2_	480±240

We envisioned that incorporation of a biotin moiety would facilitate future pull‐down experiments, so a test click reaction of **A1** with commercially available biotin‐PEG3‐azide was carried out (Scheme 3). The click reaction generated the expected tris‐triazole product **C** cleanly, as monitored by HPLC and LCMS (see Sections 2 and 5 in the Supporting Information).

Isothermal calorimetry experiments were carried out to test whether our synthesised photoaffinity probe **A1** achieved binding affinity comparable to that of similar stapled analogues (e.g., the non‐TAMRA‐labelled **A0** sequence stapled with 1.3‐diethynylbenzene; *K*_d_=6.7±2.8 nm).8b, 10 The binding affinity of **A1** for MDM2 was 18±6 nm, thus suggesting that the staple modification did not significantly impact target binding. We also synthesised an F3A negative control **B1**, which was significantly less potent as a result of mutating one of the key binding residues to alanine.

The crosslinking ability of **A1** was investigated by incubation with recombinant MDM2. Upon UV irradiation at 365 nm, successful crosslinking to MDM2 was observed by in‐gel fluorescence of the TAMRA label after SDS‐PAGE (Figure 1). **A1** crosslinking was time‐dependent over the course of an hour, whereas **B1** (F3A control peptide) showed no crosslinking to the target (Section S7).

**Figure 1:**
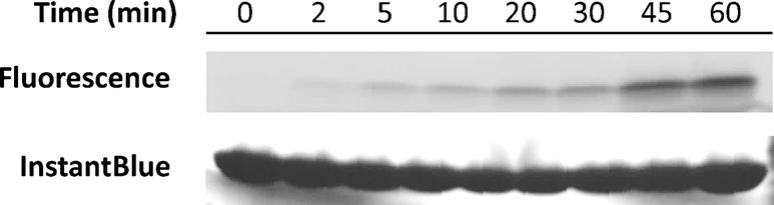
Photoaffinity labelling of MDM2 (170 μm) with **A1** (1 μm), visualised by in‐gel fluorescence and InstantBlue protein staining (note: band intensities are not directly comparable between the two images). See the Supporting Information for F3A control; full gels in Figure S 7.

In order to determine if the observed crosslinking was specific for MDM2, **A1** was incubated with mixtures of MDM2 and bovine serum albumin (BSA). Clear bands reflected preferential labelling of MDM2 by **A1** (not **B1**), and the addition of BSA did not significantly impact the ability of **A1** to crosslink to MDM2 (Figure 2).

**Figure 2:**
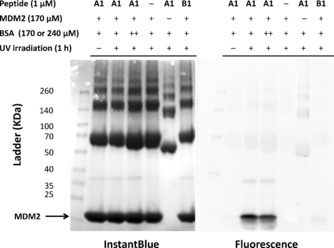
Photoaffinity labelling of MDM2 in the presence of BSA. MDM2 is preferentially labelled by peptide **A1**; control peptide **B1** does not label MDM2. BSA was at 170 μm (+) or 240 μm (++).

In summary, we designed and synthesised a novel multifunctional linker to serve as both a peptide stapling reagent and a photoaffinity probe with pulldown capability. The benzophenone linker successfully underwent copper‐catalysed double‐click stapling to generate **A1**. Subsequent reaction of the terminal alkyne on the linker with a biotinylated azide demonstrated the potential to carry out pull‐down assays with the probe. The binding affinity of the stapled probe was comparable to those of previously studied p53 stapled peptides. Finally, the probe effectively crosslinked with MDM2 after UV irradiation, and the crosslinking was specific for MDM2 over competing BSA. This methodology is currently limited to labelling purified protein and known PPIs. The next step is MDM2 labelling and pulldown in cell lysate or live cells. We envisage that this linker strategy could be applied to study other PPIs and their corresponding peptidic inhibitors.

## Experimental Section

**Linker synthesis**: 3,5‐Dibromobenzaldehyde (**2**) and ((4‐bromophenyl)ethynyl)trimethylsilane **5** were purchased from Sigma–Aldrich. 3,5‐Bis((trimethylsilyl)ethynyl)benzaldehyde (**3**)^13^ and 3,5‐diethynylbenzaldehyde (**4**)^14^ were synthesised as previously reported.

**(3,5‐Diethynylphenyl)(4‐((trimethylsilyl)ethynyl)phenyl)methanol (6)**: *n*BuLi (1.6 m in hexanes, 11.9 mL, 19.0 mmol) was added dropwise over 10 min to a stirring solution of **5** (3.44 g, 13.7 mmol) in dry THF (40 mL) at −78 °C under N_2_. The mixture was stirred for 30 min, then **4** (969 mg, 6.29 mmol) in dry THF (20 mL) was added dropwise. The reaction mixture was allowed to warm to RT and stirred for 16 h. H_2_O (30 mL) was added, and the two phases were separated. The aqueous phase was extracted with Et_2_O (3×50 mL). The combined organic extracts were dried over MgSO_4_, filtered and concentrated in vacuo. The residue was purified by column chromatography (petroleum ether/EtOAc, 20:1) to give the product (640 mg, 31 %) as a clear oil. ^1^H NMR (400 MHz, CDCl_3_): *δ*=7.50–7.49 (m, 1 H), 7.45–7.41 (m, 4 H), 7.28–7.24 (m, 2 H), 5.71 (s, 1 H), 3.09 (s, 2 H), 0.25 ppm (s, 9 H); ^13^C NMR (100 MHz, CDCl_3_): *δ*=144.1, 143.2, 143.6, 132.1, 130.4, 128.4, 126.3, 122.5, 104.7, 94.5, 82.4, 78.1, 74.8, −0.2 ppm; IR (neat): $\tilde{\nu }$ =3291, 2957, 2155, 1587, 1506, 1249, 1043 cm^−1^; HRMS (*m*/*z*) for C_22_H_20_OSi: calcd: 311.1256 [*M*+H−H_2_O]^+^, found: 311.1269.

**(3,5‐Diethynylphenyl)(4‐((trimethylsilyl)ethynyl)phenyl)methanone (1)**: A solution of **6** (205 mg, 0.627 mmol) in dry CH_2_Cl_2_ (2 mL) was added dropwise to a stirring suspension of DMP (318 mg, 0.750 mmol) in dry CH_2_Cl_2_ (2 mL) at RT under N_2_. The reaction mixture was stirred for 2 h. NaHCO_3_ (10 % aq.) saturated with Na_2_S_2_O_3_ (15 mL) was added, and the mixture was extracted with EtOAc (3×50 mL). The combined organic phase was washed with NaHCO_3_ (10 %) saturated with Na_2_S_2_O_3_ (3×50 mL), dried over MgSO_4_, filtered and concentrated in vacuo. The residue was purified by column chromatography (petroleum ether/EtOAc, 20:1) to give the product (130 mg, 64 %) as a white solid. M.p.: 109–110 °C; ^1^H NMR (400 MHz, CDCl_3_): *δ*=7.83 (d, *J*=1.5 Hz, 2 H), 7.80 (t, *J*=1.5 Hz, 1 H), 7.75–7.72 (m, 2 H), 7.61–7.58 (m, 2 H), 3.17 (s, 2 H), 0.29 ppm (s, 9 H); ^13^C NMR (100 MHz, CDCl_3_): *δ*=193.9, 138.7, 137.8, 135.9, 133.2, 131.9, 129.8, 127.9, 123.0, 103.8, 98.4, 81.5, 79.2, −0.2 ppm; IR (neat): $\tilde{\nu }$ =3280, 3065, 2959, 2156, 1656, 1599, 1311, 1221 cm^−1^; HRMS (*m*/*z*) for C_22_H_18_OSi: calcd: 327.1205 [*M*+H]^+^, found: 327.1210.

**Double‐click stapling**:8b, 10 A prepared solution of copper(II) sulfate pentahydrate (1 equiv), tris(3‐hydroxypropyltriazolylmethyl)amine (1 equiv) and sodium ascorbate (3.0 equiv) in degassed water was added to a stirring solution of the diazido peptide (1.0 equiv; 1 mL mg^−1^) and dialkynyl linker **1** (1.1 equiv) in degassed *tert*‐butanol/water (1:1) at RT under N_2_. The reaction mixture was stirred for 6 h, lypohilised and purified by HPLC to give the final stapled peptide. The reaction between **A1** (1 equiv) and biotin‐PEG3‐azide (1.2 equiv; Sigma–Aldrich) was carried out under the same click conditions.

**HPLC analysis and purification**: Analytical HPLC was performed on a 1260 Infinity LC System (Agilent Technologies) with a SUPELCOSIL ABZ+Plus column (150 mm×4.6 mm, 3 μm) and linear gradient elution (solvent A: TFA (0.05 %, *v*/*v*) in water; solvent B: TFA (0.05 %, *v*/*v*) in acetonitrile; 1 mL min^−1^). Semipreparative HPLC was performed on the 1260 Infinity with a SUPELCOSIL ABZ+Plus column (250 mm×21.2 mm, 5 μm) and linear gradient elution (solvent A: TFA (0.1 %, *v*/*v*) in water; solvent B: TFA (0.05 %, *v*/*v*) in acetonitrile; 20 mL min^−1^). HPLC was monitored by UV absorbance at 555 nm.

**Preparation of recombinant MDM2**: The expression plasmid for MDM2 (6‐125), kindly provided by Dr Anasuya Chattopadhyay (Department of Pharmacology, University of Cambridge), was transformed into *Escherichia coli* C41 competent cells.^12^ Cells were grown in 2TY medium with ampicillin at 37 °C to OD_600_=0.6, then induced with IPTG (0.5 mm) overnight at 25 °C. The cells were pelleted, resuspended in ice‐cold buffer A (Tris**⋅**HCl (50 mm, pH 8.0), NaCl (500 mm), DTT (5 mm), EDTA (1 mm), Triton X‐100 (0.1 %, *v*/*v*), EDTA‐free protease inhibitors (Roche)) and lysed with an Emulsiflex C5 homogeniser (Glen Creston). Lysate was bound onto glutathione Sepharose 4B beads (GE Healthcare) for 1 h at 4 °C. The beads were washed with buffer B (buffer A without Triton X‐100 or protease inhibitors), then cleaved on‐resin with PreScission protease (GE Healthcare) overnight at 4 °C. The cleaved protein was purified by gel filtration (HiLoad 16/60 Superdex G75; GE Healthcare). Protein identity, purity and concentration were determined by amino acid analysis.

**Isothermal calorimetry**: Calorimetric titrations were performed on a MicroCal ITC200 (Malvern Instruments, Malvern, UK). Protein and peptides were exchanged into buffer containing Na_2_HPO_4_ (50 mm), KH_2_PO_4_ (10 mm, pH 7.4), NaCl (137 mm), KCl (2.7 mm), TCEP (0.5 μm), P20 surfactant (0.005 %) and DMSO (2 %). The titration experiments were performed at 21 °C with an initial injection (0.4 μL, duration 0.8 s) followed by 19 injections (2 μL, 4 s) at 120 s spacing. For the binding assays, MDM2 (50 μm) was titrated into peptide solution (5 μm). Binding isotherms were fit by non‐linear regression with the single‐site model provided in Origin software (MicroCal, Inc. OriginLab, Northampton, MA). The stoichiometry of the interaction (N), equilibrium association constant (*K*_a_) and change of enthalpy (Δ*H*) were allowed to vary during the fitting.

**Photoaffinity labelling of recombinant MDM2**: A mixture of benzophenone stapled peptide **A1** or **B1** (0.1 nmol) and recombinant MDM2 (17 nmol) in Tris buffer (100 μL) was incubated for 15 min at RT, and then irradiated at 365 nm in a Longwave Ultraviolet Crosslinker (model CL‐1000 L; UVP, Upland, CA) for the indicated time. Irradiated samples were analysed by SDS‐PAGE on 4–20 % tricine gels (Expedeon, San Diego, CA) and visualised by in‐gel fluorescence imaging in a Typhoon FLA 9500 (555 nm; GE Healthcare) and InstantBlue protein staining (Expedeon). Binding specificity experiments with BSA (Fraction V, pH 7.0; GE Healthcare) were performed as above with the indicated concentrations of BSA in PBS buffer.

## Supporting information

As a service to our authors and readers, this journal provides supporting information supplied by the authors. Such materials are peer reviewed and may be re‐organized for online delivery, but are not copy‐edited or typeset. Technical support issues arising from supporting information (other than missing files) should be addressed to the authors.

Supplementary
