# Supplementary material for: Development of a Multifunctional Benzophenone Linker for Peptide Stapling and Photoaffinity Labelling
Source: Chembiochem. 2016 Mar 16;17(8):689–92. doi: 10.1002/cbic.201500648 (PMC4862033; doi:10.1002/cbic.201500648)
Supplement: Supplementary file 1 — Supplementary [file CBIC-17-689-s001.pdf]

CHEMBIOCHEM

Supporting Information

Development of a Multifunctional Benzophenone Linker for Peptide Stapling and Photoaffinity Labelling

Yuteng Wu+,[a] Lasse B. Olsen+,[a] Yu Heng Lau+,[a] Claus Hatt Jensen,[a] Maxim Rossmann,[b] Ysobel R. Baker,[a] Hannah F. Sore,[a] Súil Collins,[a] and David R. Spring*[a]

cbic_201500648_sm_miscellaneous_information.pdf

Supporting information

1. **Peptide synthesis**
2. **Peptide LCMS data**
3. **Analytical and chromatographic methods**
4. **Isothermal calorimetry**
5. **Pull-down handle**
6. **NMR Spectra for linker 1 and synthetic intermediates**
7. **Full gels of photoaffinity labeling**

1. Peptide synthesis:

Peptide synthesis was carried out on solid-phase using an Fmoc-protecting group strategy on a CEM Liberty Automated Microwave Peptide Synthesiser using Merck Rink Amide MBHA resin LL (0.38 mmol/g). All peptide couplings were performed with Fmoc-protected amino acids (5 equiv) in DMF, HATU (5 equiv) in DMF, and N,N-diisopropylethylamine (10 equiv) in NMP. Arginine was coupled using double couplings for 15 min each without microwave irradiation. All other amino acids were coupled using single couplings with 25 W power at 75 ^{o}C for 15 min. Fmoc deprotection was carried out with 20 % piperidine in DMF, using 45 W power at 75 ^{o}C over 3 min. N-terminal capping with TAMRA was performed manually by treating the resin-bound peptide with TAMRA (6 equiv), HATU (6 equiv) and DIPEA (12 equiv) in DMF for 3 h. Cleavage was achieved with a cocktail of trifluoroacetic acid (92.5%), triisopropylsilane (2.5%), water (2.5%), dichloromethane (2.5%) for 2 h. The cleavage solution was then evaporated under a stream of nitrogen. The crude residue was triturated with diethyl ether prior to purification by semi-preparative HPLC.

2. Peptide LCMS data

Table S.2. LCMS data for unstapled and stapled peptides. Calculated m/z ratios are for $[M+2H]^{2+}$.

Peptide	Mass	m/z found	m/z calcd
A0	2239.1	1120.7	1120.6
B0	2163.1	1082.9	1083.0
A1	2493.4	1248.2	1248.1
B1	2417.3	1210.2	1210.1
C	2937.9	1470.3	1470.2

Peptide **A0**

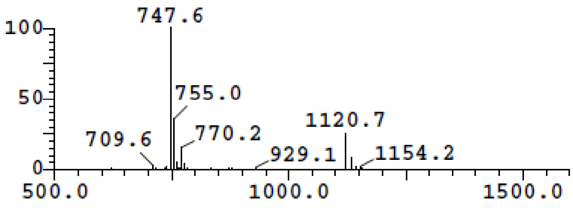

Peptide **B0**

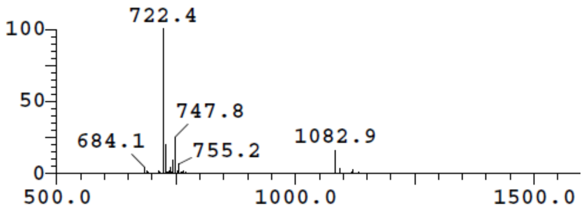

Peptide **A1**

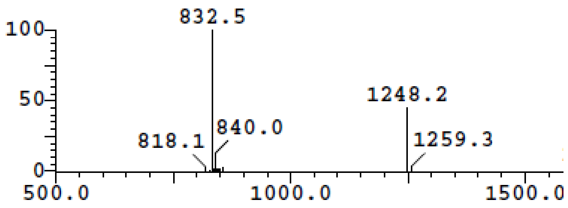

Peptide **B1**

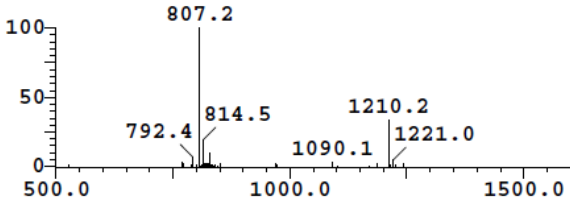

Peptide **C**

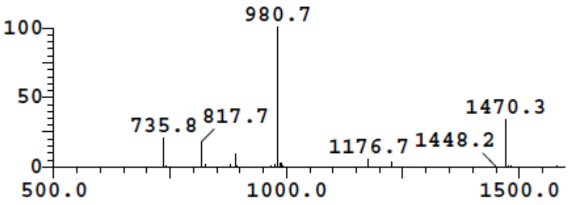

TMS-protected stapled peptide intermediate

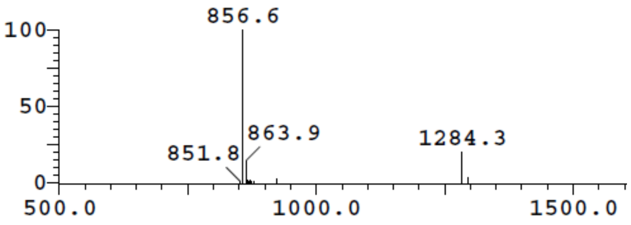

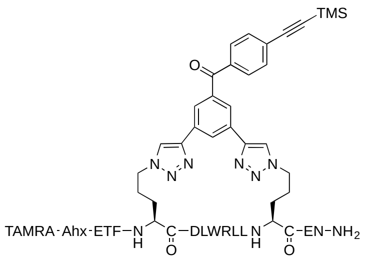

m/z: 1284.3

3. Analytical and chromatographic methods

Liquid chromatography-mass spectrometry (LCMS) was run on an Agilent 1200 series LC with an ESCi Multi-Mode ionisation waters ZQ spectrometer (LC system : solvent A: 10 mM ammonium acetate + 0.1% formic acid in water; solvent B: 95% acetonitrile + 5% water + 0.05% formic acid; column: Supelcosil ABZ+PLUS column (33 mm × 4.6 mm,3 μm); gradient: 0.0-0.7 min: 0% B, 0.7-4.2 min: 0- 100% B, 4.2-7.7 min: 100% B, 7.7-8.5 min: 100-0% B), or a waters ACQUITY H-Class UPLC with an ESCi Multi-Mode ionisation Waters SQ Detector 2 spectrometer (LC system : solvent A: 2 mM ammonium acetate in water/acetonitrile (95:5); solvent B: 100% acetonitrile; column: AQUITY UPLC CSH C18, 2.1*50 mm, 1.7 µm, 130 Å; gradient: 5-95% B over 3 min with constant 0.1% formic acid). Retention times are reported to the nearest 0.1 min.

Infra-red spectra were recorded neat on a Perkin Elmer Spectrum One FT-IR Spectrometer fitted with an attenuated total reflectance (ATR) sampling accessory; absorption maxima are reported in wavenumbers (cm^{-1}) and quoted to the nearest 1 cm^{-1}.

High resolution mass spectrometry (HRMS) was recorded on a Waters LCT Premier Time of Flight mass spectrometer.

4. Isothermal calorimetry

K_d values – **A1**: 18 ± 6 nM; **B1**: 480 ± 240 nM

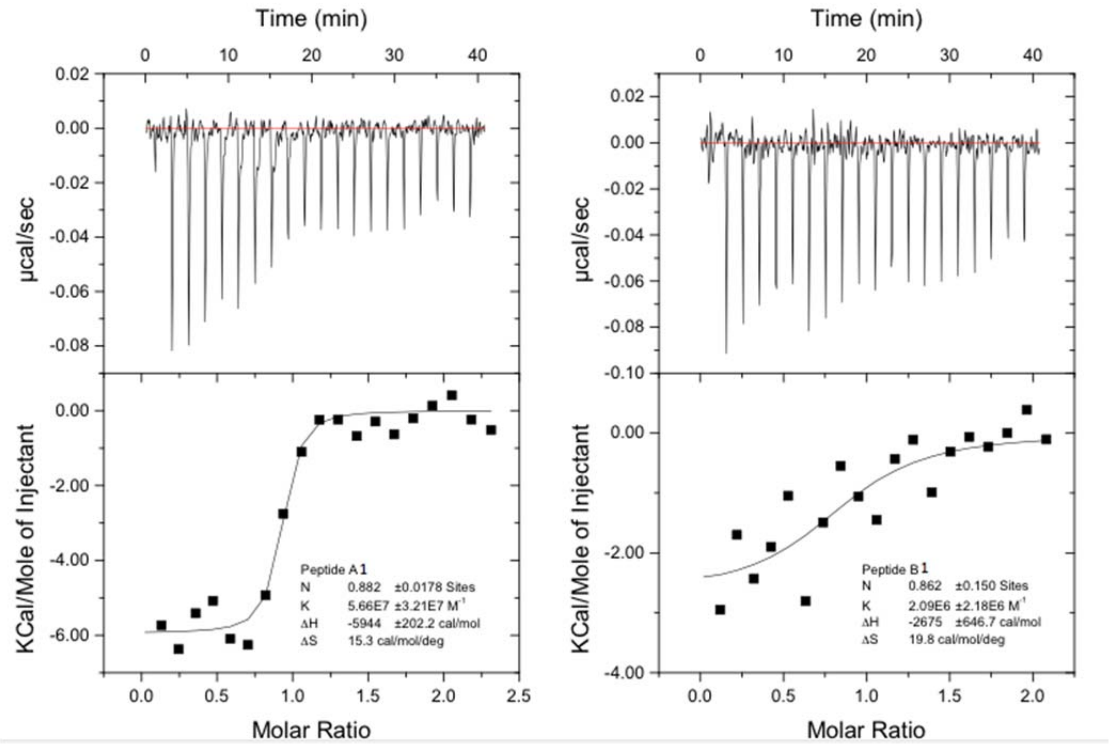

Figure S4.1 ITC data for peptides **A1** and **B1**.

Control ITC experiments

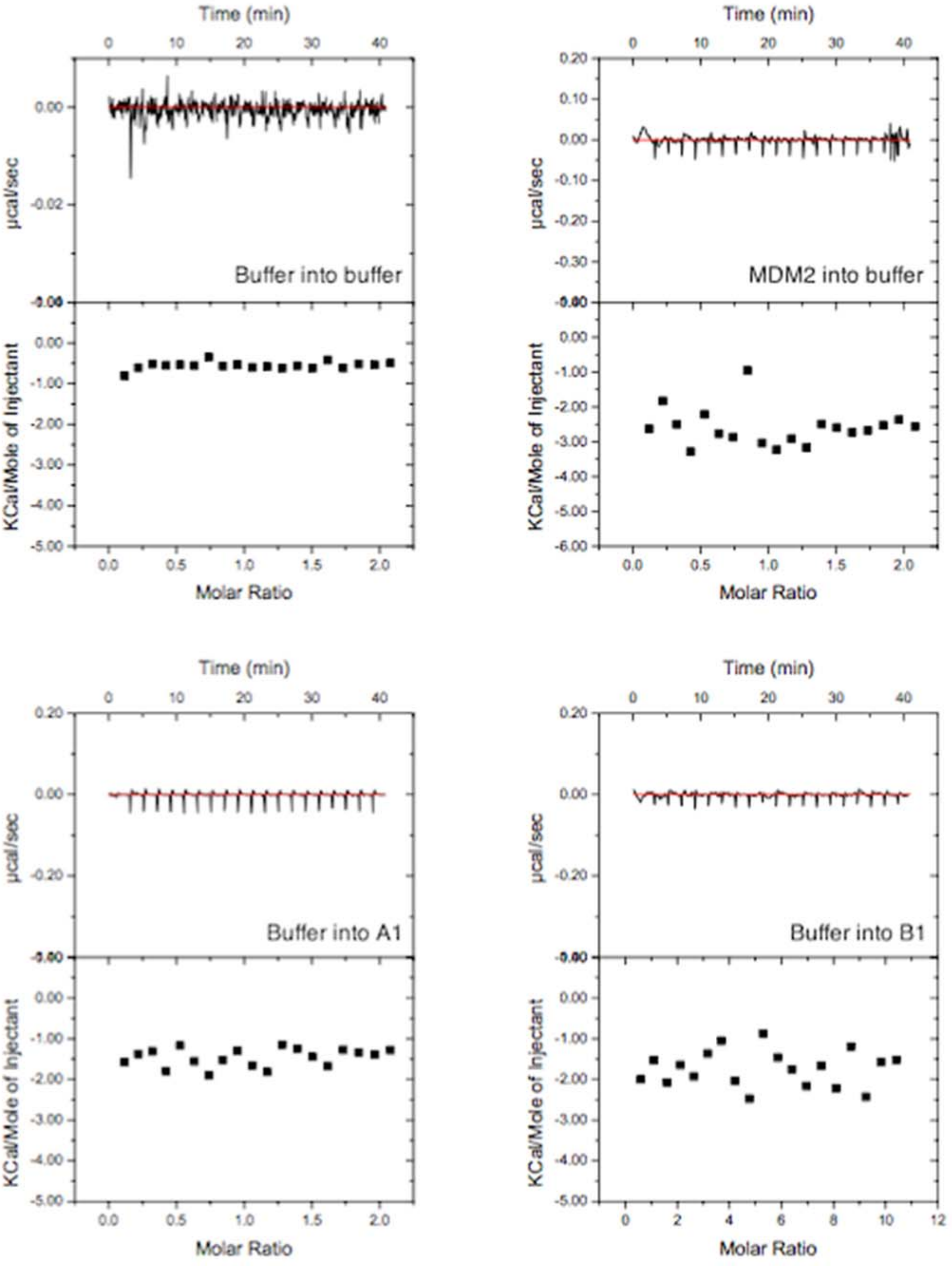

Figure S4.2 ITC data for buffer into buffer, MDM2 into buffer, buffer into **A1** and buffer into **B1**. Buffer contains 50 mM Na_2HPO_4, 10 mM KH_2PO_4, pH 7.4, 137 mM NaCl, 2.7 mM KCl, 0.5 µM TCEP, 0.005 % P20 surfactant and 2% DMSO.

5. Pull-down handle

Figure S.5. HPLC chromatographs of starting peptide **A1** (top), and the crude reaction mixture after click reaction with biotin-PEG3-azide to give the tris-triazole product **C** (bottom), monitored at 555 nm.

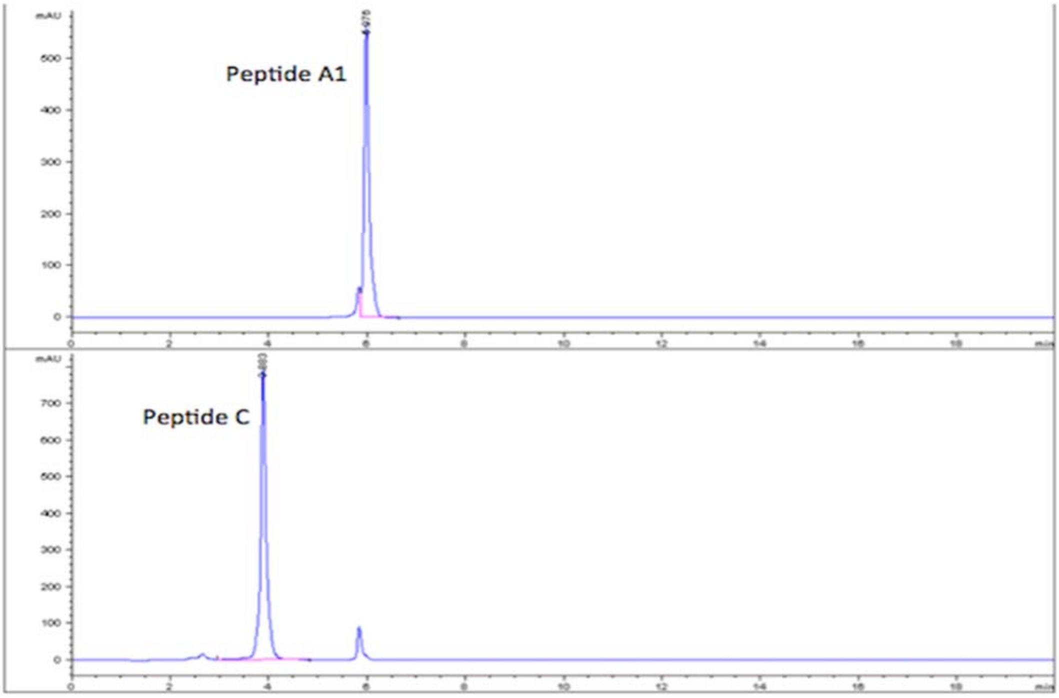

6. NMR Spectra for linker 1 and synthetic intermediates

3,5-Bis((trimethylsilyl)ethynyl)benzaldehyde **3**

^{1}H NMR

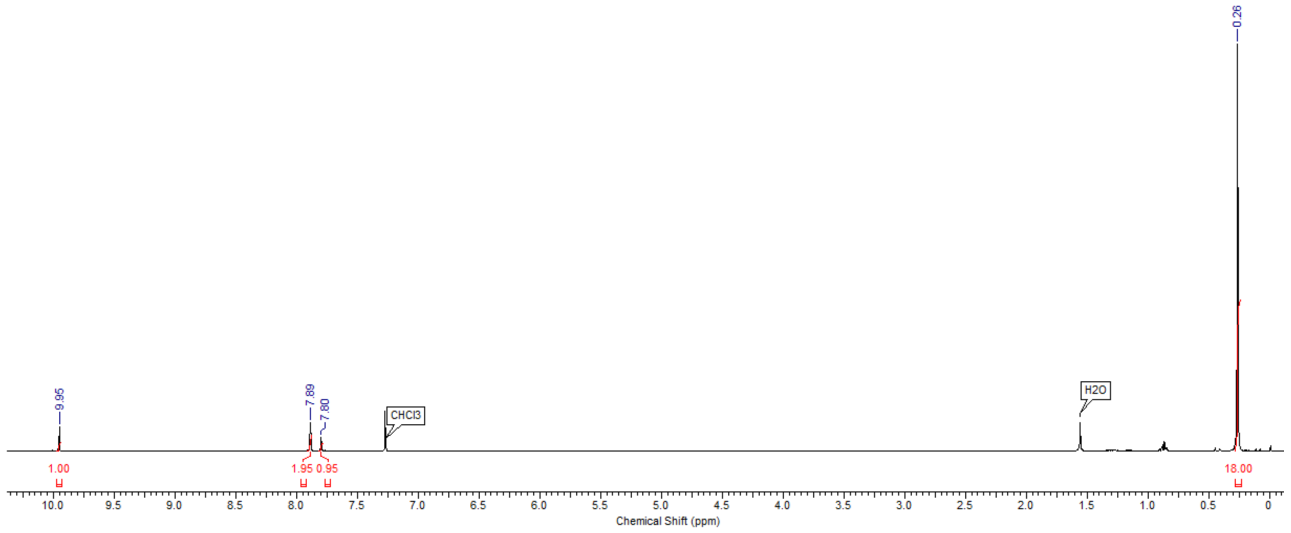

^{13}C NMR

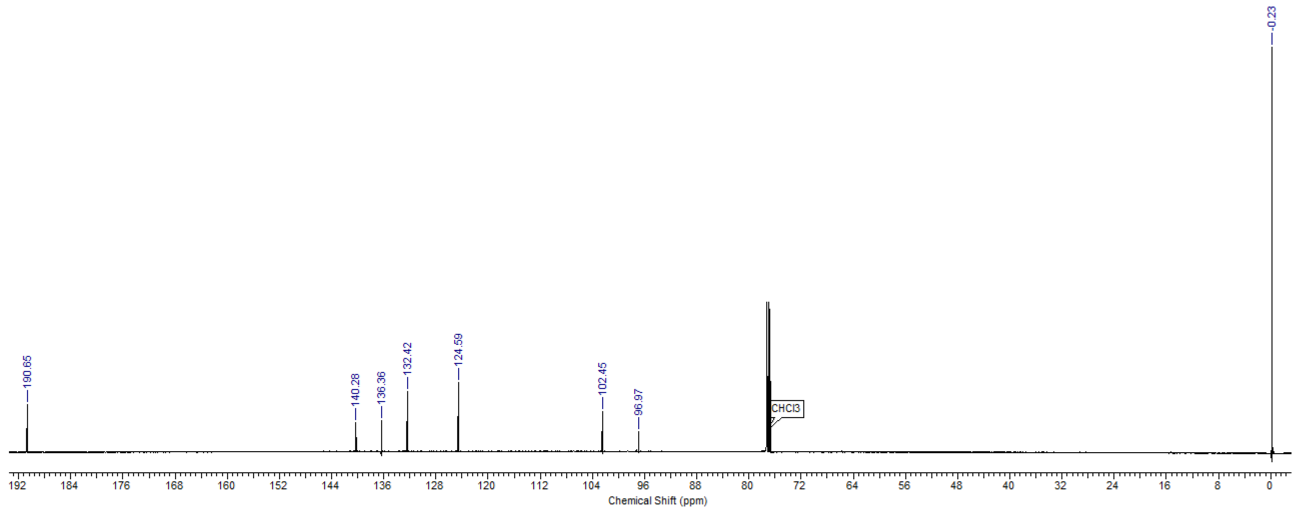

3,5-Diethynylbenzaldehyde **4**

^{1}H NMR

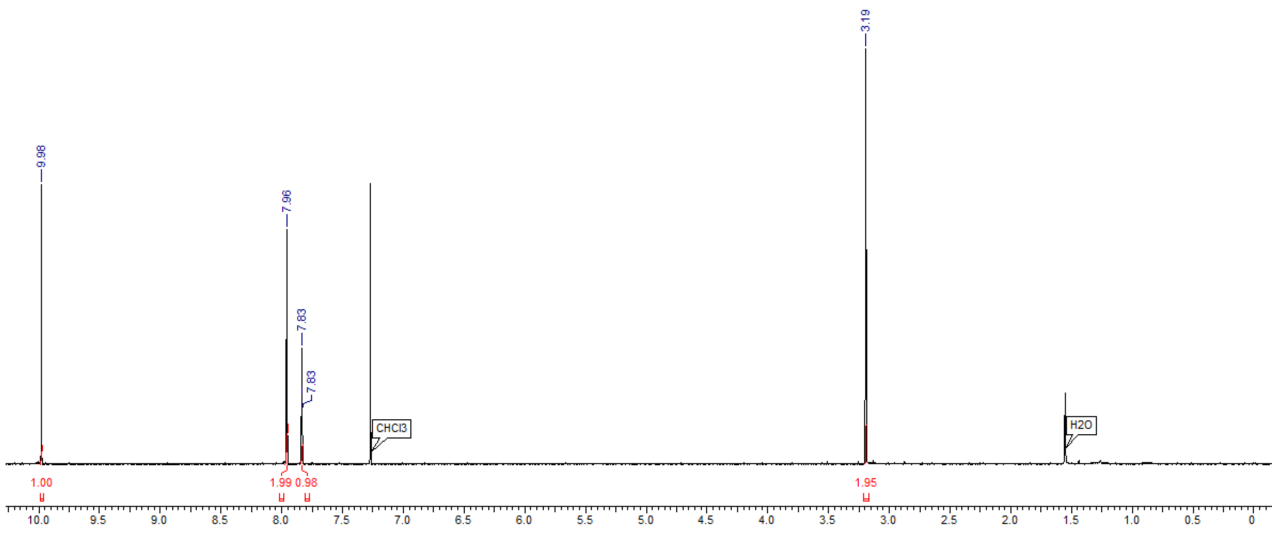

^{13}C NMR

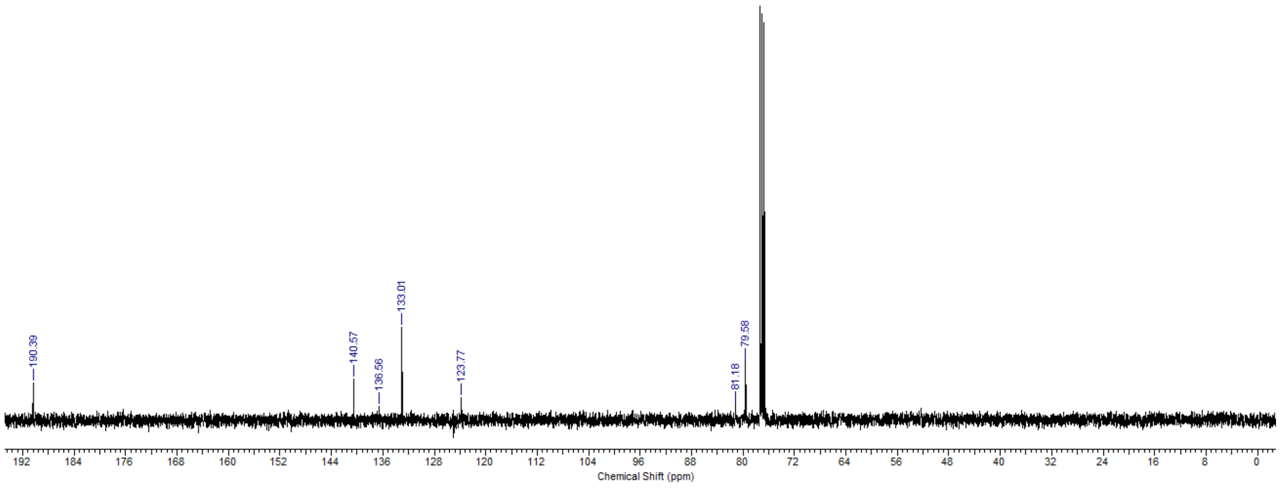

(3.5-diethynylphenyl)(4-((trimethylsilyl)ethynyl)phenyl)methanol **6**

^{1}H NMR

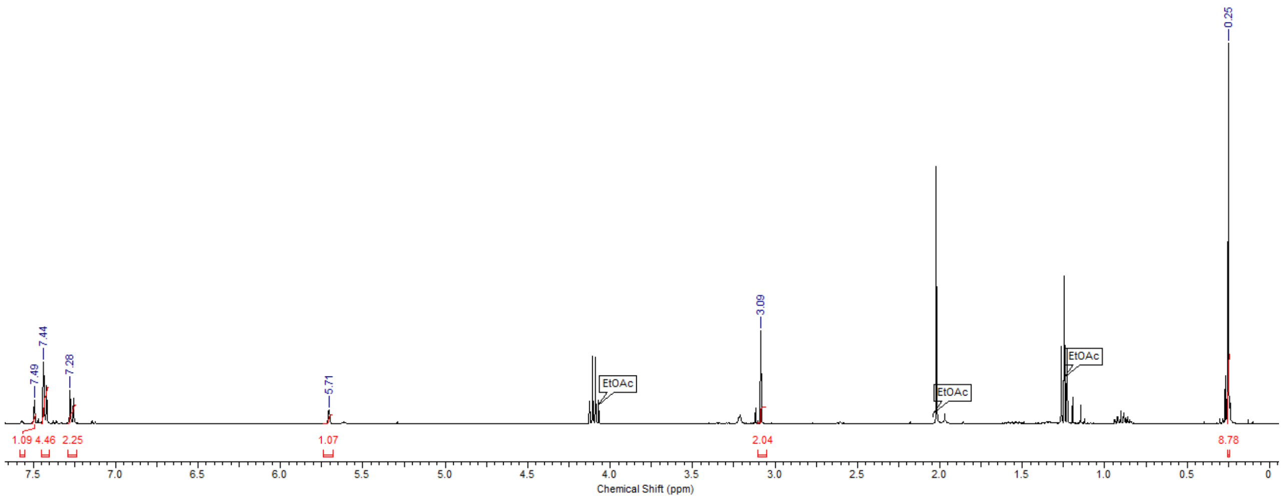

^{13}C NMR

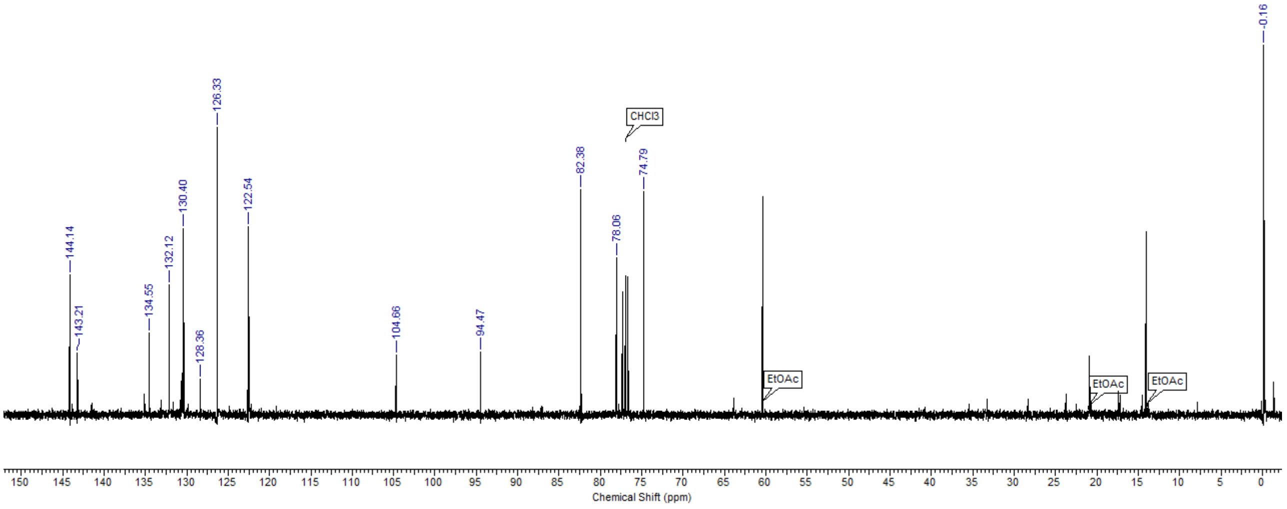

(3,5-Diethynylphenyl)(4-((trimethylsilyl)ethynyl)phenyl)methanone **1**

^{1}H NMR

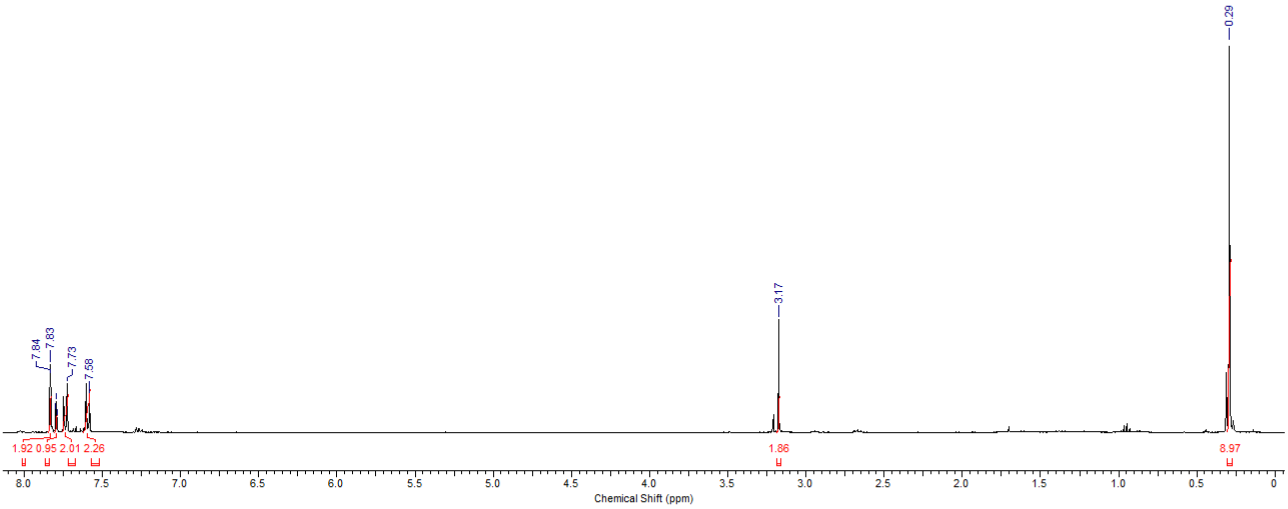

^{13}C NMR

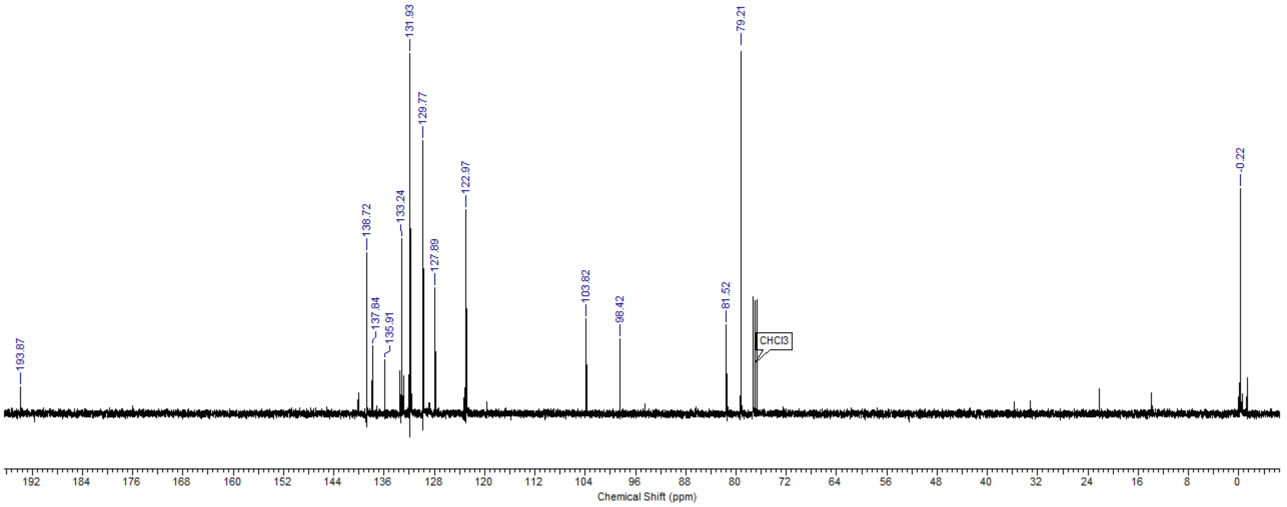

7. Full gels of photoaffinity labeling

Figure S7.1. Photoaffinity labeling of MDM2 with **A1**, visualised by in-gel fluorescence and InstantBlue stain.

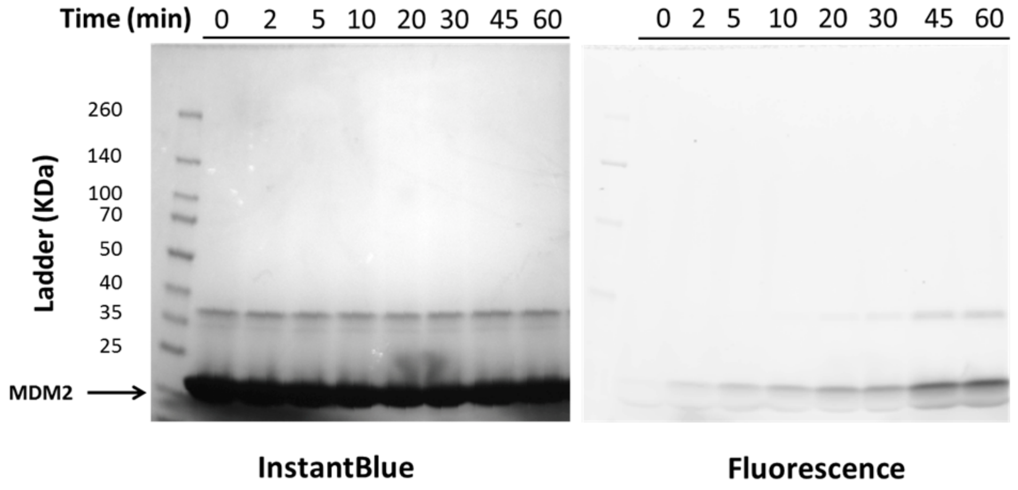

Figure S7.2. Photoaffinity labeling of MDM2 with **B1**, visualised by in-gel fluorescence and InstantBlue stain.

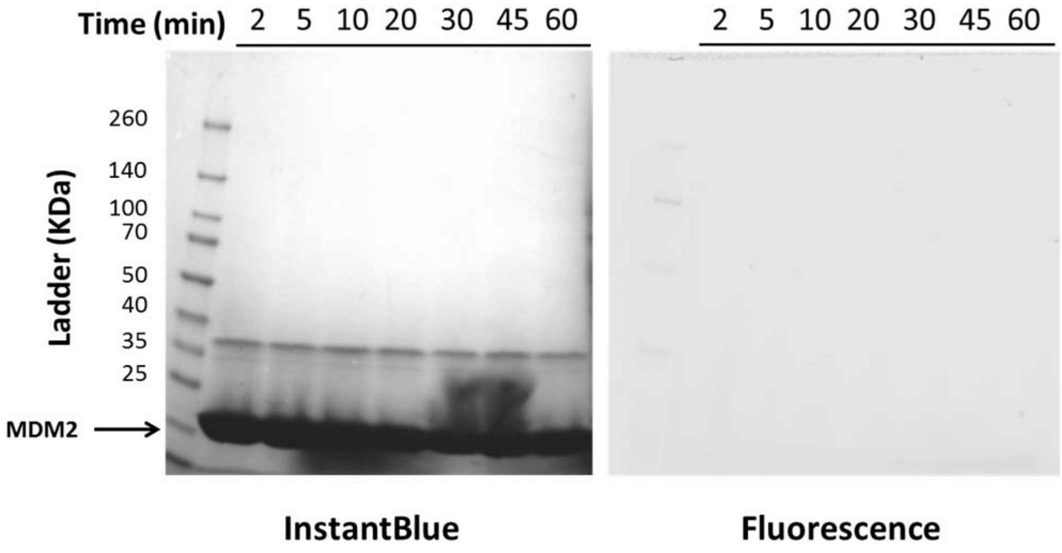

The minor impurity at ~35 kDa corresponds to uncleaved GST-MDM2 fusion protein. Amino acid analysis indicates that the amino acid composition is within 5% of the expected values for MDM2 (6-125).
